# Perceived satisfaction with care, service atmosphere and mood fluctuations among patients with schizophrenia spectrum disorder in Italian mental health supported accommodations: a multicenter study

**DOI:** 10.1186/s12888-026-08365-7

**Published:** 2026-10-21

**Authors:** Alessandra Martinelli, Elena Toffol, Donato Martella, Martina Carnevale, Stefano Calza, Giovanni de Girolamo

**Affiliations:** 1 Unit of Epidemiological Psychiatry and Digital Mental Health, IRCCS Istituto Centro San Giovanni di Dio Fatebenefratelli, Brescia, Italy; 2 Department of Public Health, Clinicum, Faculty of Medicine, University of Helsinki, PO Box 20 (Tukholmankatu 8B), Helsinki, 00014 Finland; 3 Unit of Biostatistics and Bioinformatics, Department of Molecular and Translational Medicine, University of Brescia, Brescia, Italy

**Keywords:** Patient satisfaction, Mood, Schizophrenia spectrum and other psychotic disorders, Residential facilities, Ecological momentary assessment

## Abstract

**Background:**

Supported Accommodations (SA) are central to Italy’s community-based mental health care for individuals with schizophrenia spectrum disorders (SSD), offering structured, recovery-oriented environments. While symptom severity remains important, care environment and satisfaction strongly influence well-being. Mood fluctuations, a key aspect of daily functioning, have been underexplored in SA. Using the Experience Sampling Method (ESM), this study aims to examine the associations between perceived ward atmosphere, care satisfaction, and momentary mood in SA residents with SSD.

**Methods:**

As part of the DiAPAson project, 68 residents with SSD were assessed also using ESM. Additional measures included psychopathology (BPRS, BNSS), functioning (SLOF, WHODAS 2.0), perceived ward atmosphere (WAS-P) and service satisfaction (VSSS-32). Descriptive analyses were conducted, and a structural equation model examined mood in relation to clinical, demographic, and environmental factors.

**Results:**

Residents with SSD (29.4% female; mean age = 42.8 years) were mostly single (80.9%) and unemployed (79.4%), with a mean disorder duration of 19.2 years. Clinical assessments indicated moderate symptom severity and functioning. Structural equation modelling showed excellent fit (CFI = 1.000; RMSEA = 0.000; SRMR = 0.060). The latent variable Env-Perc, combining service satisfaction (VSSS-32) and perceived ward atmosphere (WAS-P), was the only significant predictor of momentary mood (β = 0.344, 95%CI: [0.084;0954], *p* = 0.033).

**Conclusions:**

Perceived satisfaction with care and service atmosphere in SA contribute to promoting positive mood among individuals with SSD.

**Trial registration:**

ISRCTN registry ID ISRCTN21141466, registration date 17 February 2020.

## Background

Residential care is a cornerstone of community-based mental health services, offering stable housing and individualized support for people with severe mental disorders [1]. Among European countries, service availability and access to residential care vary significantly. According to WHO reports [2–4], important differences persist across European regions, with many Eastern European countries, generally characterized by lower levels of economic development, still relying more on centralized, large-scale institutional models, whereas Western European countries have progressively developed more decentralized, community-based services [5, 6]. Italy, along with England, have developed a structured system of Supported Accommodations (SA), grounded in the principles of the progressive care model [7, 8]. This model enables individuals to move from high-support to lower-support or independent living settings as they develop functional and social skills, aligning with recovery-oriented goals such as autonomy, empowerment, and societal participation. While evidence suggests that this model can support recovery-oriented outcomes [7, 9, 10], it may also present challenges, including the need for multiple residential transitions during the recovery process and the risk of prolonged lengths of stay in SA [7, 9, 10].

SA are specifically designed for individuals with severe mental disorders, particularly those with schizophrenia spectrum disorders (SSD), who typically present with persistent symptoms, high clinical needs, and marked psychosocial impairments. They provide structured environments that facilitate recovery by promoting autonomy, helping residents relearn daily living skills, encouraging social reintegration, and supporting engagement in meaningful adult roles [9, 11, 12]. Before the Italian psychiatric reform and deinstitutionalization process, these individuals often experienced long-term institutionalization [13–15]. In Italy, psychiatric care was fundamentally restructured with the approval of Law 180 (the so-called *Basaglia Law*) in 1978, which marked a shift from hospital-based care to a community-oriented mental health system, mandating the gradual closure of psychiatric hospitals and the development of community-based services [16].

SA represent a central component of Italy’s long-term mental health care system. In this context, SA are community-based residential facilities that provide a combination of housing, clinical support, and rehabilitative interventions, with varying levels of staffing intensity and therapeutic input. These services range from highly supported settings with 24-hour staff presence to more independent living arrangements with lower levels of support, reflecting different stages of recovery [17, 18].

According to Italian national guidelines [18], SA are classified into five main types based on two key dimensions: rehabilitation intensity and care intensity [19]. These include high-support facilities (SRP1 and SRP2), intermediate-support facilities (SRP3.1), and lower-support or supported housing solutions (SRP3.3). Additionally, SRP3.2 facilities are designed for individuals with severe but stable mental health disorders who may require long-term support and are less likely to transition to more independent settings [18].

This structured system is intended to support a progressive care pathway, enabling individuals to move from higher to lower levels of support as they develop functional and social skills [10, 17]. SA are managed by both non-profit and for-profit private providers, with accommodation and care costs fully covered by the Italian National Health Service [18].

Understanding the outcomes of individuals living in SA is essential not only from a clinical and research standpoint but also from the perspective of those with lived experience. Their insights are critical in guiding both practice and policy. While symptom severity remains an important clinical marker, features of the care environment—especially the ward atmosphere—play a fundamental role in shaping the well-being of both residents and staff. The concept of ward atmosphere represents the social climate of the ward, and encompasses the emotional and physical qualities of a care setting; as such, it significantly affects how care is experienced, influencing patient satisfaction, treatment adherence, and staff morale [20–22]. Ward atmosphere is commonly measured with the Ward Atmosphere Scale (WAS) [20], which consists of the three dimensions of relationship, personal growth and system management.

Residents’ perceived satisfaction with care is another essential aspect of quality in mental health services. Satisfaction is linked to the quality of housing and interpersonal relationships. Even when adjusting for demographics and levels of functioning, positive perceptions of the living environment and social interactions remain strong predictors of overall satisfaction with care [23].

Mood fluctuations are another critical dimension of resident well-being. To explore these dynamics in everyday life, the Experience Sampling Method (ESM) offers a powerful and ecologically valid tool. ESM collects real-time data on individuals’ emotions, thoughts, and activities in natural settings [24]. While ESM has been widely applied to assess mood and symptom fluctuations in people with SSD [25], its use in evaluating mood fluctuations in SA remains limited [26–28].

The present study aims to investigate, for the first time, the associations between perceived global ward atmosphere measured with the WAS, satisfaction with care, and momentary mood among individuals with SSD living in SA across Italy.

## Methods

### Design setting and recruitment

This research is a component of the national initiative titled “DAily time use, Physical Activity, quality of care and interpersonal relationships in patients with Schizophrenia spectrum disorders (DiAPAson)” [29]. The DiAPAson project involved 20 Departments of Mental Health (each encompassing a different number of SA) and 17 private SA situated in various regions across Italy.

The inclusion criteria comprised individuals aged between 20 and 55 years, a DSM-5 diagnosis of SSD [26], and proficiency in the Italian language for effective participation in research interviews. Exclusion criteria encompassed severe cognitive impairments (e.g., Mini-Mental State Examination corrected score < 24), inability to provide informed consent, a recent (last six months) DSM-5 diagnosis of substance use disorder [30], a cerebrovascular/neurological disease, and a history of clinically significant head injury.

In the participating SA, facility leaders generated an alphabetical list of SSD patients on a specific day, and residents were consecutively invited to join the study until the recruitment goal was met. Between October 2020 and October 2021, 340 residents with SSD were enrolled from 98 SA (81 belonging to the 20 participating Departments of Mental Health, and 17 from the private sector) across Italy (12.8±5.7 residents per SA). Six patients (1.8%) were excluded due to severe cognitive deficits, and 21 (6.2%) withdrew after providing informed consent. Consequently, 313 patients were included in the original DiAPAson project, with each SA contributing an average of 3.5 ± 2.6 residents. Thus, we recruited approximately 27.0% of patients from each participating SA based on the total number of occupied beds.

**Fig. 1:**
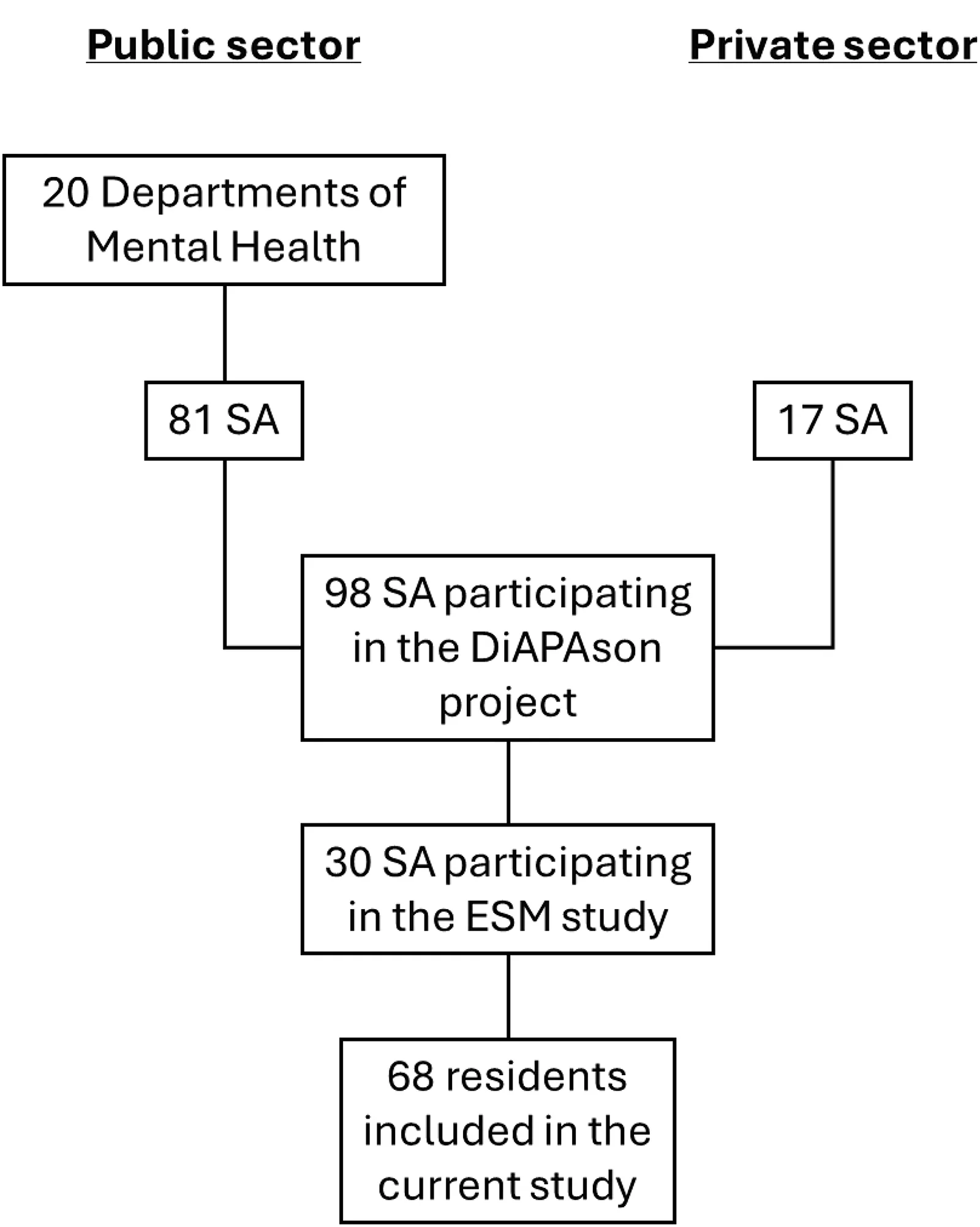
Flowchart of the recruitment process

Because of logistical and financial constraints, the ecological ESM study could only be conducted in a subset of SA (*N* = 30), which accepted to carry out this part of the project having more human resources to manage this specific research activity, and which were included in the current study. In these facilities 68 residents (21.7%) were assessed using the ESM (Fig. 1).

### Assessments

Sociodemographic data (age, gender, years of schooling, marital and working status) and clinical data on the patient’s condition (length of illness, overall duration of hospitalizations, total number of hospitalizations in the last year, and time in the SA) were obtained from clinical records. Questionnaires were filled in by the treating physicians and/or by the residents with the help of research assistants, as appropriate, before starting the ESM. The monitoring ESM phase was preceded by a 30-minute briefing session delivered by research assistants, who provided detailed instructions on the procedures.

The *severity of psychopathology* was assessed using the 24-item Brief Psychiatric Rating Scale (BPRS) [31, 32]. Ratings for BPRS items were conducted on a 7-point Likert scale, ranging from 1 (no symptoms) to 7 (extremely severe symptoms), in response to the question “Please enter the score for the term which best describes the patient’s condition” and were categorized into five groups (depression/anxiety (e.g.,”Worry, fear, or over-concern for present or future”), excitement (e.g., “Heightened emotional tone, agitation, increased reactivity”), positive symptoms (e.g., “Perceptions without normal external stimulus correspondence”), negative symptoms (e.g., “Reduced emotional tone, apparent lack of normal feeling or involvement”), and cognitive symptoms (e.g., “Confusion or lack of proper association for person, place or time”).

To evaluate the *severity of negative symptoms* specifically, the Brief Negative Symptom Scale (BNSS) [33, 34] was employed. BNSS items (e.g., “intensity of pleasure during activities”) were rated on a 6-point Likert scale, spanning from 0 (no symptoms) to 6 (severe symptoms), and encompassed assessments of blunted affect, alogia, asociality, anhedonia, and avolition. In both the BPRS and BNSS, higher mean scores indicated a greater severity of symptomatology.

The investigation into the *perceived ward atmosphere* by both patients (WAS-P) and healthcare staff (WAS-T) utilized the “Ward Atmosphere Scale” (WAS) developed by Moos and Houts in 1968 [20]. Only the patients’ perception (WAS-P) was considered in the current study. This instrument consists of 100 dichotomous (Yes/No) items that capture various aspects of the ward. The higher-order dimensions of the scale are relational, personal growth, and system maintenance, each associated with ten corresponding subscales (e.g., “Patients put a lot of energy into what they do around here”, “Doctors have very little time to encourage patients”). These subscales, including involvement, support, spontaneity, autonomy, practical orientation, personal problem orientation, anger and aggression, order and organization, program clarity, and staff control, assess specific aspects of the ward environment. Each item is scored as 0 or 1 (Y/N), contributing to a subscale score ranging from 0 to 10. Subscale averages are then calculated to provide an overall assessment. The WAS scale, widely employed in clinical settings and program evaluations, allows for the comparison of perceptions between patients and healthcare staff [21, 35].

*Satisfaction with care* was assessed using the Verona Service Satisfaction Scale (VSSS-32) which comprises 32 items (e.g., “How satisfied are you with the ability of staff to listen/understand your problems?”), each rated on a five-point Likert scale (1 = not at all satisfied; 5 = extremely satisfied). The items are grouped into 7 subscales (overall satisfaction, professionals’ skills and behaviour, efficacy, types of intervention, information, access, relatives’ involvement) [36].

*Psychosocial functioning* was evaluated using the Specific Levels of Functioning Scale (SLOF), which measures the level of support a participant requires to carry out various tasks (e.g., “Engages in activities without prompting”). The scale includes 43 items, each rated on a 5-point Likert scale, yielding a total score between 43 and 215. Higher scores indicate better psychosocial functioning [37].

*Functioning and disability* were assessed with the WHO Disability Assessment Schedule (WHODAS 2.0), consisting of 12 items (e.g., “In the last 30 days how much difficulty did you have in concentrating on doing something for ten minutes?”) rated on a 5-point Likert scale (0 = no difficulty; 4 = extreme difficulty), where higher scores denote greater levels of disability [38].

Following clinical assessment and questionnaire completion, *Experience Sampling Method* was employed to assess daily activities, social interactions and mood with a questionnaire administered on a smartphone-based application, developed ad hoc for the project, over a seven-day period. In details, each participant received semi-randomised notifications (i.e., randomly sent within the scheduled time slots) across eight time slots (8–10 a.m., 10 a.m.–12 p.m., 12 p.m.–2 p.m., 2–4 p.m., 4–6 p.m., 6–8 p.m., 8–10 p.m., 10 p.m.–12 a.m.), each followed by a reminder notification after 15 minutes. The participants had a maximum of 30 min to reply. Mood was assessed with the question “Right now, I feel …”, with possible answer options being “happy, sad, tired, relaxed, nervous, quiet, full of energy”.

### Statistical analysis

Data are presented as mean, standard deviation, median, minimum and maximum for continuous variables, and as count and percentage for categorical variables. In this analysis, a structural equation model (SEM) was developed to explore the factors influencing mood. This model incorporates observed variables and the individual’s perception of their environment as a latent construct. Univariate normality was assessed using the Shapiro–Wilk test, along with skewness and kurtosis indices, and by visually inspecting histograms and Q–Q plots. Three variables—Mood, Age, and the total score on the BPRS—displayed statistically significant but minimal deviations from normality. In contrast, the other continuous variables exhibited distribution patterns consistent with the assumption of normality. The screening for univariate and multivariate outliers did not reveal any influential outliers. Given the small sample size and the minimal departures from normality, non-parametric bootstrap standard errors were employed in the SEM to ensure robust estimation and inference, despite potential violations of distributional assumptions. The model is based on the hypothesis that a combination of demographic factors, clinical variables, and environmental perception shapes mood. Individual’s environmental perception is a latent variable called Env-Perc (environment perception), indicating a psychological dimension that cannot be directly measured but is inferred from observable indicators. In this case, two self-report instruments were used: the VSSS-32 total score for perceived social support and the WAS-P total score for environmental satisfaction. Env-Perc, derived from the combination of VSSS-32 and WAS-P, along with other variables (age, sex, illness duration, BPRS total score, BNSS total score and SLOF total score), was examined in relation to mood. Statistical analyses were performed using R software, version 4.3.1. A significance level of 0.05 was adopted for all statistical tests.

## Results

### Sociodemographic and clinical characteristics of the sample

The sample included 68 participants, comprising 20 females (29.4%) and 48 males (70.6%). Their socio-demographic and clinical characteristics are reported in Table 1. Briefly, their mean age was 42.8 years (SD = 10.5). Most of them were single (80.9%), while 8.8% were married or living together, and 10.3% were divorced or widowed. On average, participants had 11.3 years of education (SD = 3.5). Regarding employment status, 16.2% were employed, 4.4% were studying, and 79.4% were unemployed. The average duration of illness was 19.2 years (SD = 10.2). In terms of lifetime psychiatric hospitalization, 54.4% had spent between 1 and 5 years in psychiatric facilities, while 45.6% had been hospitalized for more than 5 years. Additionally, 35.3% reported being smokers. In terms of clinical assessment, the mean score on BPRS (Cronbach’s alpha = 0.80) was 47.3 (SD = 12.9), and the BNSS (Cronbach’s alpha = 0.91) had a mean score of 23.3 (SD = 14.2). The SLOF (Cronbach’s alpha = 0.92), which evaluated functioning, showed a mean score of 175.7 (SD = 20.9), while the WHODAS 2.0 (Cronbach’s alpha = 0.71), which assessed disability, had a mean score of 33.3 (SD = 25.2). Service satisfaction, measured by VSSS-32 (Cronbach’s alpha = 0.89), yielded a total mean score of 3.8 (SD = 0.5), while WAS-P (Cronbach’s alpha = 0.87), which assessed perceived ward atmosphere by patients, had a mean score of 56.8 (SD = 10.3).

**Table 1:** Socio-demographic and clinical characteristics of residents (*N* = 68)

Variables	N (%)
**Sex, n (%)**	
*Female*	20 (29.4%)
*Male*	48 (70.6%)
**Age, n (%)**	
*Mean (SD)*	42.8 (10.5)
*Median (Min- Max)*	45.5 (21.0–56.0)
**Marital status, n (%)**	
*Single*	55 (80.9%)
*Married/cohabiting*	6 (8.8%)
*Divorced/ widowed*	7 (10.3%)
**Education years**	
*Mean (SD)*	11.3 (3.5)
*Median (Min- Max)*	11.0 (2.0–21.0)
**Working status, n (%)**	
*Working*	11 (16.2%)
*Studying*	3 (4.4%)
*Not working*	54 (79.4%)
**Illness duration**	
*Mean (SD)*	19.2 (10.2)
*Median (Min- Max)*	20.0 (1.0–40.0)
**Lifetime duration of psychiatric hospitalization (years), n (%)**	
*1–5 years*	37 (54.4%)
*>5 years*	31 (45.6%)
**Smoking Status, n (%)**	
*Yes*	24 (35.3%)
*No*	44 (64.7%)
**BPRS**	
*Mean (SD)*	47.3 (12.9)
*Median (Min- Max)*	46.0 (27.0–78.0)
**BNSS**	
*Mean (SD)*	23.3 (14.2)
*Median (Min- Max)*	22.0 (0.0–55.0)
**SLOF**	
*Mean (SD)*	175.7 (20.9)
*Median (Min- Max)*	174.0 (125.0–212.0)
**WHODAS 2.0**	
*Mean (SD)*	33.3 (25.2)
*Median (Min- Max)*	25.0 (2.0–101.0)
**WAS-P ***Mean (SD)*	
*Involvement*	49.1 (10.1)
*Support*	7.0 (2.4)
*Spontaneity*	50.2 (8.9)
*Autonomy*	45.1 (5.5)
*Practical Orientation*	55.0 (8.7)
*Personal Problem Orientation*	51.0 (8.7)
*Anger and Aggression*	43.2 (8.8)
*Order and Organisation*	58.6 (8.7)
*Program Clarity*	59.3 (11.1)
*Staff Control*	48.4 (9.6)
*Total mean score*	56.8 (10.3)
***VSSS-32 ****Mean (SD)*	
*Overall satisfaction*	4.1 (0.9)
*Professionals’ skills and behaviour*	4.0 (0.7)
*Information*	3.7 (1.1)
*Access*	4.2 (0.8)
*Efficacy*	3.7 (0.9)
*Types of intervention*	3.8 (0.4)
*Relatives’ involvement*	3.3 (1.3)
*Total mean score*	3.8 (0.5)

BPRS = Brief Psychiatric Rating Scale; BNSS = Brief Negative Symptom Scale; SLOF = Specific Levels of Functioning Scale; WAS-P = Ward Atmosphere Scale-patient version; VSSS-32 = Verona Service Satisfaction Scale 32 item

### Results of structural equation model

**Fig. 2:**
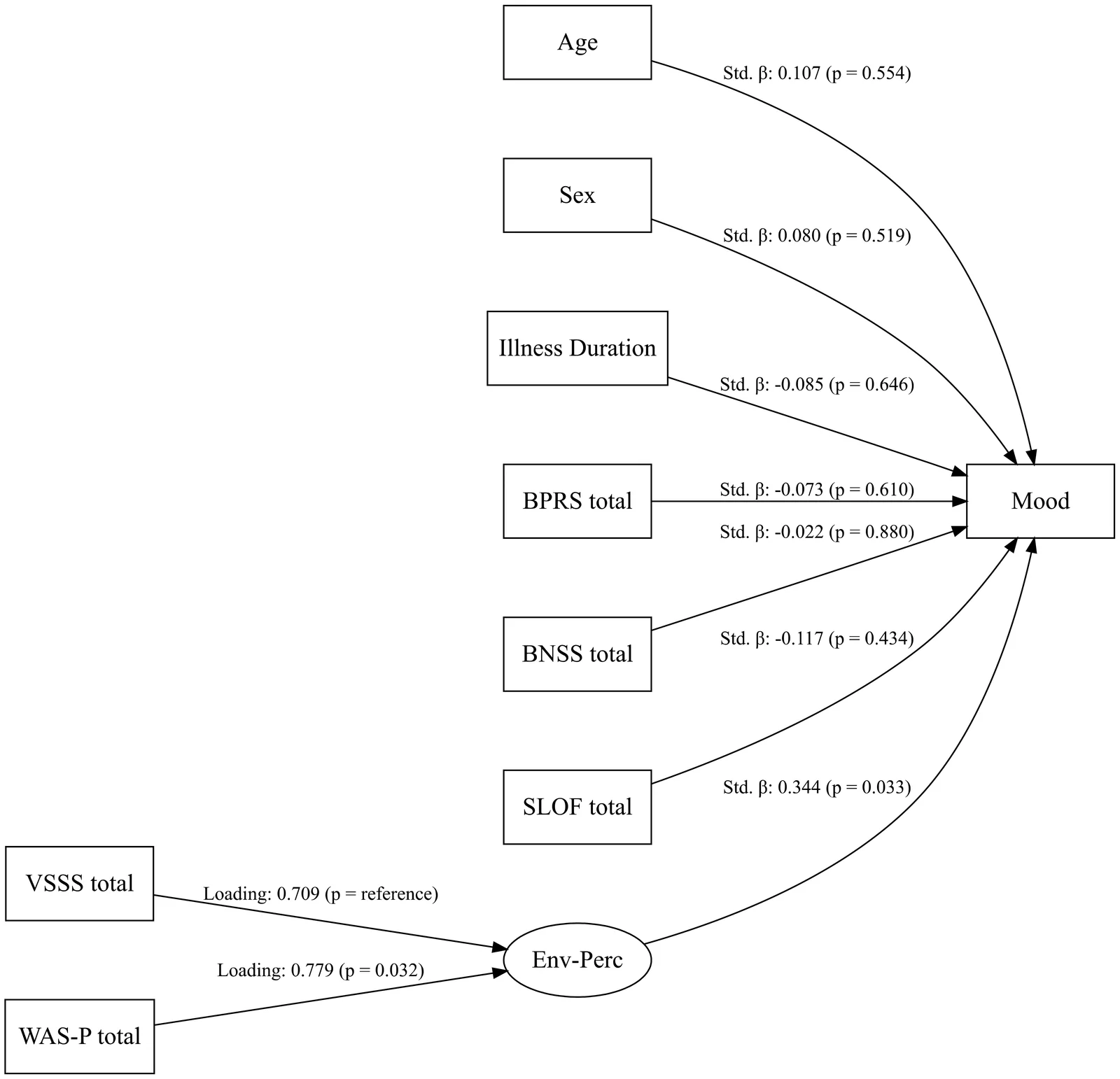
Overall picture of the SEM model with coefficients. Reference denotes the indicator used for scaling the latent variable, with its loading fixed at 1 to ensure model identification

The SEM developed to investigate the determinants of mood revealed that environmental perception, represented as a latent construct (Env-Perc), was the only statistically significant predictor of mood among the variables examined. This latent factor, derived from the total scores of the VSSS-32 and the WAS-P, demonstrated strong and significant factor loadings. Specifically, the WAS-P total score had a high standardized loading of 0.779 (*p* = 0.032), while the VSSS-32 total was fixed at 1.00 for identification purposes and showed a standardized loading of 0.709, confirming their contributions to the underlying construct of environmental perception (Fig. 2). The VSSS-32 total score was fixed at 1.00 to establish a scale for the latent construct. This approach aligns with standard procedures in SEM, where one indicator is constrained to 1. This constraint helps set the metric for the latent variable without influencing the estimation or interpretation of the other parameters.

The model fit indices indicated an excellent fit to the data: the chi-square test was non-significant (χ^2^ = 8.072, *p* = 0.779), and all fit indices surpassed conventional thresholds, with a Comparative Fit Index (CFI) of 1.000, a Root Mean Square Error of Approximation (RMSEA) of 0.000 (90% CI: 0.000–0.088), and a Standardized Root Mean Square Residual (SRMR) of 0.060.

Among the predictors considered, only Env-Perc was significantly associated with mood (β = 0.344, 95%CI: [0.084;0954], *p* = 0.033), suggesting that individuals who reported more positive perceptions of their environment tended to experience better mood states. In contrast, demographic variables and clinical scales showed no significant relationship with mood (Table 2).

**Table 2:** Results of the SEM model

Variable	β*	95%CI	p-value
Env-Perc	0.344	0.084; 0.954	**0.033**
Age	0.107	−0.350; 0.586	0.554
Sex	0.080	−0.392; 0.777	0.519
Disease duration	−0.085	−0.496; 0.304	0.646
BPRS total	−0.073	−0.330; 0.165	0.610
BNSS total	−0.022	−0.322; 0.282	0.880
SLOF total	−0.117	−0.354; 0.194	0.434

*Standardised regression coefficient

Bold *p*-values indicate statistical significance (*p* < 0.05)

## Discussion

This study is the first to explore the relationship between perceived ward atmosphere, satisfaction with care, and momentary mood among individuals with SSD residing in SA across Italy. Our findings provide valuable insights into how environmental perceptions influence emotional well-being in this population.

Using SEM, we identified that only the latent variable—Env-Perc—which captures the shared construct of perceived environment, integrating the two validated self-report measures VSSS-32 and the WAS-P - emerged as the significant predictor of momentary mood, while individual demographic and clinical variables—such as age, gender, illness duration, symptom severity, and social functioning—showed no significant effects. Although the model explained only 14% of the variance in mood, this finding highlights the role of environmental perceptions in shaping emotional states, independent of traditional clinical predictors.

These results align with existing literature indicating that satisfaction with care and therapeutic ward atmosphere are closely tied to mood and overall psychological well-being in people with SSD [39, 40]. Studies have shown that higher satisfaction with care is associated with better quality of life and fewer negative emotional states [41], while unmet needs in areas like autonomy, accommodation, or social activities are linked to poorer mood and less engagement in meaningful daily activities [42].

The impact of satisfaction with care goes beyond service quality; personal factors such as self-efficacy, insight and social support have also been identified as contributors to satisfaction and mood insight [43, 44]. Similarly, facilities that foster recovery-oriented practices and promote human rights tend to support greater well-being and emotional stability among residents [45–47]. Opportunities for meaningful engagement—through structured daily activities, peer interaction, or work-like experiences—have also been associated with improved mood and satisfaction [48–51].

Interestingly, literature also points to discrepancies between staff and resident perceptions of needs, with staff often overestimating met needs compared to residents’ self-assessments [42, 52–55]. This misalignment may contribute to dissatisfaction and negative mood and underscores the importance of incorporating the patient’s voice into care planning and service evaluation.

The relatively modest proportion of mood variance explained (14%) indicates that while the perceived environment plays a role, other elements not addressed in this study—such as trauma history, stigma, cognitive distortions, or neurobiological variables—likely contribute to emotional well-being. Future research should incorporate a broader range of psychosocial and clinical factors to build more comprehensive predictive models.

## Strengths and limitations

A key strength of this study lies in the consistent application of the ESM across all participants, using real-time data collection to capture momentary experiences. Standardized and validated clinical instruments were employed to ensure methodological rigor and comparability.

However, several limitations should be noted. The findings may not be generalizable to individuals with diagnoses other than SSD or to those with significant cognitive impairment or to settings others than SA. Data were collected during the Sars-CoV-2 pandemic, a period marked by significant disruptions to daily routines, which may have influenced participants’ mood and environmental perceptions. A further limitation of this study is the lack of complete information regarding the specific type of SA where participants lived, preventing a more detailed characterization of residential settings and related analyses. Because we wanted to examine the relationship between global patients’ perception of ward atmosphere, rather than on specific dimensions, and momentary mood, only the average WAS-P total score was used in this study. This approach may have missed potentially meaningful patterns, and future studies should consider employing subscale scores to examine the role of different dimensions of ward climate.

## Conclusions

The findings of our study suggest that perceived satisfaction with care in SA for individuals with SSD is crucial for maintaining a stable and positive mood. Interventions targeting ward atmosphere and service satisfaction—such as fostering supportive staff-patient relationships, addressing unmet needs, and providing structured daily activities—may significantly improve mood outcomes in individuals with SSD residing in SA. Encouraging a recovery-oriented ethos and tailoring care environments to meet residents’ subjective needs could yield substantial benefits in terms of emotional well-being and engagement in rehabilitation.

## Data Availability

De-identified data from this study are available in a public archive: dataset referring to this manuscript is published with restricted access on Zenodo platform and accessible at this link: https://zenodo.org/records/17034126

## Abbreviations

BNSS: Brief Negative Symptom Scale
BPRS: Brief Psychiatric Rating Scale
CFI: Comparative Fit Index
ESM: Experience Sampling Method
RMSEA: Root Mean Square Error of Approximation
SA: Supported Accommodations
SEM: structural equation model
SLOF: Specific Levels of Functioning Scale
SRMR: Standardized Root Mean Square Residual
SSD: schizophrenia spectrum disorders
VSSS-32: Verona Service Satisfaction Scale
WAS: Ward Atmosphere Scale
WHODAS 2.0: WHO Disability Assessment Schedule
